# tDCS Stimulation of the dlPFC Selectively Moderates the Detrimental Impact of Emotion on Analytical Reasoning

**DOI:** 10.3389/fpsyg.2018.00568

**Published:** 2018-04-19

**Authors:** Bastien Trémolière, Véronique Maheux-Caron, Jean-François Lepage, Isabelle Blanchette

**Affiliations:** ^1^EA 7352 Chrome, Université de Nîmes, Nîmes, France; ^2^Département de Psychologie, Université du Québec à Trois-Rivières, Trois-Rivières, QC, Canada; ^3^Département de Pédiatrie, Université de Sherbrooke, Sherbrooke, QC, Canada

**Keywords:** emotion, transcranial direct current stimulation, dorsolateral prefrontal cortex, analytical reasoning, working memory

## Abstract

There is evidence of a detrimental effect of emotion on reasoning. Recent studies suggest that this relationship is mediated by working memory, a function closely associated with the dorsolateral prefrontal cortex (dlPFC). Relying on transcranial direct current stimulation (tDCS), the present research explores the possibility that anodal stimulation of the dlPFC has the potential to prevent the effect of emotion on analytical reasoning. Thirty-four participants took part in a lab experiment and were tested twice: one session using offline anodal stimulation (with a 2 mA current stimulation applied to the left dlPFC for 20 min), one session using a control (sham) stimulation. In each session, participants solved syllogistic reasoning problems featuring neutral and emotionally negative contents. Results showed that anodal stimulation diminished the deleterious effect of emotion on syllogistic reasoning, but only for a subclass of problems: problems where the conclusion was logically valid. We discuss our results in the light of the reasoning literature as well as the apparent variability of tDCS effects.

## Introduction

There is a great deal of evidence that emotion affects a vast range of cognitive activities. Among these, reasoning has been given some research attention (Oaksford et al., 1996; Blanchette and Richards, 2004; Blanchette, 2006; Blanchette and Leese, 2011; Jung et al., 2014), to show that it is greatly shaped by emotion (for a review, see Blanchette et al., 2017). In these studies, emotion is manipulated either by making the context emotional, or by using emotional contents in the reasoning problems. Manipulating emotional content involves framing reasoning problems with (most of the time negative) emotional words. Consider, for instance, the two following problems:

(1)
  - There are old people who are retired;
  - No retired person is an astronaut.
  - Therefore, there are old people who are not astronauts.
(2)
  - There are victims who are ugly;
  - No ugly person is raped.
  - Therefore, there are victims who are not raped.

When asked to indicate whether the conclusion follows logically from the premises, people experience greater difficulty in correctly solving problem (2) than problem (1), even though the structure of the two problems is identical. The emotionally negative content present in problem (2) impacts people's performance. Recent data suggest that the effect of emotion on reasoning is related to cognitive load: additional cognitive resources are required to process the emotional information, even though this dimension is actually irrelevant to correctly solve the problem (Trémolière et al., 2016). In other words, emotion is thought to affect reasoning by burdening working memory.

Neuroimaging research has highlighted the physiological underpinnings of working memory. A large amount of research has shown the critical importance of the dorsolateral prefrontal cortex (dlPFC) in cognitive activities involving working memory. Lesions to the dlPFC impact performance on working memory tasks in non-human primates (Jacobsen and Nissen, 1937; Bauer and Fuster, 1976). Analogous effects have recently been observed in human beings (Müller et al., 2002). Thus, there is important evidence that dlPFC plays an important role in working memory (for reviews, see Wager and Smith, 2003; Owen et al., 2005).

Modern neurostimulation tools make it possible to draw inference about the contribution of specific brain regions to high order cognitive processes. Recent research has shown that the dlPFC activity could be modulated by tools such as transcranial magnetic stimulation (TMS) or transcranial direct current stimulation (tDCS), among others. In the past 15 years, an important number of studies have investigated the effect of tDCS stimulation of the dlPFC on different working memory tasks. For instance, online anodal stimulation of the dlPFC, in which tDCS is applied during the execution of a working memory task enhances performance on a forward-digit span task (Andrews et al., 2011) and on a complex verbal problem-solving task (the Remote Associates Test; Cerruti and Schlaug, 2009). It also enhances accuracy (Ohn et al., 2008) and decreases reaction time on an N-back task (Mulquiney et al., 2011; Teo et al., 2011). Protocols involving offline stimulations, when tDCS is applied before the task, also show promising results. Anodal stimulation of the left dlPFC prior to the task increases accuracy in an N-back task (Zaehle et al., 2011; Hoy et al., 2013), a digit span test, a visuospatial attention test, and a Stroop task (Jeon and Han, 2012). These results provide evidence for the effect of tDCS on activities related to working memory, either with online or offline stimulations (for an exhaustive review, see Tremblay et al., 2014).

In addition to its cognitive effects, tDCS of the prefrontal cortex can also have an impact on affective processes. It has been observed that anodal stimulation of the left dlPFC has a positive impact on emotion regulation (Peña-Gómez et al., 2011; Feeser et al., 2014; Salehinejad et al., 2017), and that it decreases the perceived unpleasantness of emotionally negative pictures (Boggio et al., 2009; Maeoka et al., 2012).

Thus, there is evidence that anodal tDCS of the left dlPFC might be associated with enhanced working memory and emotion regulation. This leads to specific predictions regarding the detrimental effect of emotion on reasoning: anodal stimulation of the left dlPFC, if it increases working memory capacities and/or decreases aversive reaction to emotionally negative stimuli, may reduce the commonly observed detrimental effect of emotion on analytical reasoning. This is an important issue, as people regularly reason about emotional materials. No research has yet explored the effect of tDCS on analytical and emotional reasoning. The present study addresses these issues directly.

## Method

### Participants and design

The 34 participants (26 women, mean age = 23.71 years, SD = 8.67) were recruited on campus at the Université du Québec à Trois-Rivières. On the basis of a self-report questionnaire, we included only participants who were right-handed, healthy, who did not suffer from migraine, chronic pain, psychiatric or neurological disease, who did not take psychotropic medication, and who were not pregnant. Each participant provided informed consent. Participants came to the lab twice: in one session they were subjected to an offline anodal stimulation (activation of the dlPFC), in the other session they were subjected to a control sham stimulation (also prior to performing the task). In each session, after the stimulation was done, participants were instructed to solve 16 syllogistic reasoning problems. This reasoning task was performed after a working memory task (results reported in another paper), which was completed during the stimulation (online stimulation^1^). The following features of the syllogisms were manipulated: validity (valid vs. invalid problems), believability (believable vs. unbelievable problems), and emotionality of the content (neutral vs. emotionally negative content). Two sets of problems were designed (one for each session). The order of the sessions (stimulation, sham) and sets of problems were counterbalanced across participants.

### Material

#### tDCS stimulation

The anodal electrode was placed over the left dlPFC (F3 site of the 10–20 EEG system) while the cathodal electrode was placed over the right supraorbital area. Size of electrodes was 35 cm^2^, a size that has been demonstrated to maximize the effects of stimulations (Ho et al., 2016). The anodal condition involved a direct current stimulation of 2 mA that was applied for 20 min, at a current density of 0.057 mA/cm^2^ (ramp up for 30 s to reach 2 mA, that level being then kept constant until the end of the stimulation). Current density was zero in the sham condition. A 30-s ramp-up/ramp-down was used at the beginning and at the end of the sham stimulation. This stimulation protocol has been shown to be indistinguishable from anodal stimulation to the participants (Ambrus et al., 2012). Mean delay between the first and the second session was 7.5 days (SD = 2.2; Min = 5; Max = 14).

#### Reasoning task

Our analytical task was a classic syllogistic task featuring belief bias, first introduced by Evans et al. (1983) and used subsequently in numerous investigations (Goel and Dolan, 2003; De Neys, 2006). The reasoning problems were presented using Eprime.

We manipulated validity (i.e., whether the conclusion follows logically from the premises or not) and believability (i.e., whether the conclusion is believable or not). This led to four types of problems (valid-believable; valid-unbelievable; invalid-believable; invalid-unbelievable). For instance, an example of a valid and believable problem reads:

(3)
  - There are hotels that are renovated;
  - No renovated building is deserted.
  - Therefore, there are hotels that are not deserted.

Importantly in relation to our hypotheses, half of these problems featured emotionally negative contents, as in problem (2) presented in the introduction, while the other half featured neutral contents, as in (1,3). Two sets of 16 problems were used and counterbalanced across the stimulation conditions. Participants scored 1 when they correctly solved the problem and 0 when they incorrectly solved the problem. Accuracy was transformed in percentages. Response times were also recorded.

Emotionality of the syllogisms was pretested using an independent sample of 30 Quebec participants who evaluated the emotional intensity of the problems, using a scale ranging from 1 (Not at all emotional) to 7 (Intensely emotional). Results showed that the emotional problems (M = 4.74, SD = 1.24) were rated as more emotional than the neutral problems (M = 2.03, SD = 1.03), *t*_(29)_ = 10.65, *p* < 0.001. This difference was significant within each set of syllogisms (all *p*s < 0.001).

## Data analysis

Data were analyzed by means of a repeated measures ANOVA, where validity (valid vs. invalid), believability (believable vs. unbelievable), emotion (neutral vs. emotion), and stimulation (tdcs vs. sham) were entered as independent variables and where the dependent measures were accuracy and response times for the reasoning task. Statistical analyses were conducted using SPSS software. An a priori power analysis conducted with Gpower (Erdfelder et al., 1996) indicated that a sample size of 36 would be sufficient to detect a significant interaction effect between emotion and tDCS with a power of 0.95 and an alpha of 0.05, based on an effect size of 0.25.

## Results

### Accuracy

Descriptive statistics for each stimulation condition and problem type are reported in Table 1. Repeated measures ANOVA showed that validity affected accuracy, $F_{\left(1,\;33\right)}\;=\;64.21,p<0.001,\eta_{p}^{2}=0.66$, with participants more accurate on valid problems (M = 87.13; SD = 12.59) than on invalid problems (M = 50.18; SD = 22.22). No other main effects were detected (all *F*s < 0.41, all *p*s > 0.29). A validity × believability interaction was significant, $F_{\left(1,33\right)}=30.25,p<0.001,\eta_{p}^{2}=0.48$. Pairwise comparisons showed that believability affected valid problems, *t*_(33)_ = 3.94, *p* < 0.001, Cohen's *d* = 0.78, to a slightly lesser extent than for invalid problems, *t*_(33)_ = 4.97, *p* < 0.001, Cohen's *d* = 0.91. The expected emotion × stimulation interaction was not detected, $F_{\left(1,\;33\right)}=0.01,p=0.93,\eta_{p}^{2}<0.001$.

**Table 1:** Accuracy means (and standard deviations) for each stimulation condition and problem type.

			**tDCS**	**Sham**
Neutral problems	Valid	Believable	94.12 (16.35)	97.06 (11.94)
		Unbelievable	79.41 (30.45)	79.41 (32.84)
	Invalid	Believable	41.18 (37.88)	35.29 (35.95)
		Unbelievable	67.65 (32.29)	60.29 (36.47)
Emotional problems	Valid	Believable	98.53 (08.58)	89.71 (20.52)
		Unbelievable	82.35 (27.20)	76.47 (30.74)
	Invalid	Believable	39.71 (36.47)	45.59 (41.50)
		Unbelievable	57.35 (37.20)	54.41 (31.06)

**Figure 1:**
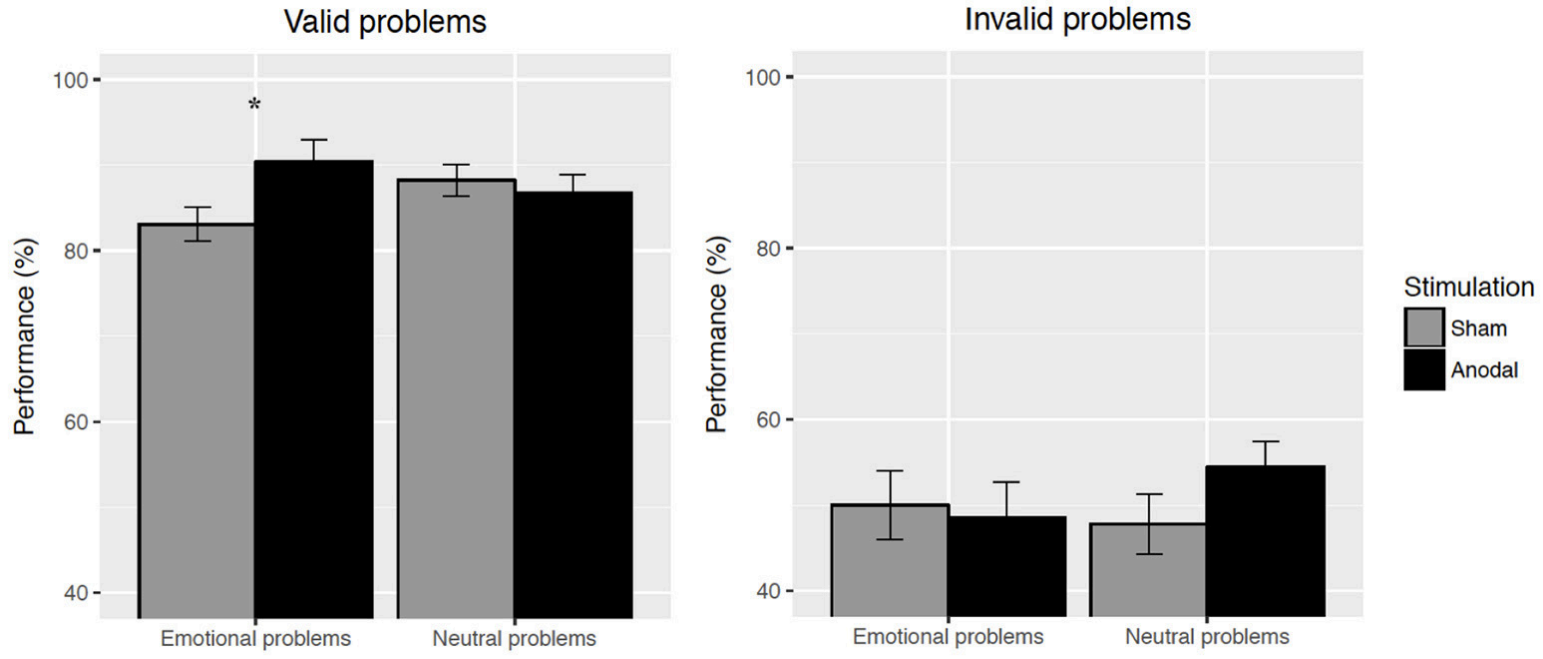
Effects of stimulation on accuracy, as a function of emotion, for valid and invalid problems separately. Error bars indicate standard error of the mean. * indicates a significant difference between anodal and sham stimulation for valid emotional problems.

Importantly in regard to our current purpose, a three-way validity × emotion × stimulation condition interaction was observed, $F_{\left(1,\;33\right)}=6.15,p=0.018,\eta_{p}^{2}=0.16$^2^ (see Figure 1 for a visual description of that interaction). We decomposed the interaction into two emotion × stimulation condition interactions, for the valid and invalid problems separately. For valid problems, no main effect was detected (all *F*s < 1.93, all *p*s > 0.17). The emotion x stimulation condition interaction was significant, $F_{\left(1,\;33\right)}=4.70,p=0.038,\eta_{p}^{2}=0.13$. Pairwise comparisons showed that anodal stimulation led to a greater accuracy on emotional problems compared to sham stimulation, *t*_(33)_ = 2.26, *p* = 0.03, Cohen's *d* = 0.46, while it had no effect for neutral problems, *t*_(33)_ = 0.57, *p* = 0.57, Cohen's *d* = 0.09. No main effect or interaction were observed for invalid problems (all *F*s < 0.1.60, all *p*s > 0.22).

Finally, a three-way validity × believability × emotion interaction fell short of significance, $F_{\left(1,\;33\right)}=2.30,p=0.14,\eta_{p}^{2}=0.07$. Because that interaction is peripheral to our main purpose to explore the interaction between stimulation condition and emotion, we do not analyze it further.

### Response times

As a next step of analysis, we explored participants' response times. Values above ±2 SD from the mean were excluded and replaced by the mean of the participant. Participants were faster on valid problems (in milliseconds; M = 12,082, SD = 6,511) than on invalid problems (M = 16,381, SD = 8,298), $F_{\left(1,\;33\right)}=34.47,p<0.001,\eta_{p}^{2}\;=\;0.51$. Also, participants took longer on emotional problems (M = 15,082, SD = 7,383) than on neutral problems (M = 13,381, SD = 7,378), $F_{\left(1,\;33\right)}=7.38,p=0.01,\eta_{p}^{2}=0.18$.

A trend for believability × stimulation condition interaction was observed, $F_{\left(1,\;33\right)}=2.73,p=0.11,\eta_{p}^{2}=0.08$. Pairwise comparisons showed that tDCS tended to decrease response times on unbelievable conclusions (M_*tDCS*_ = 13, 000, SD_*tDCS*_ = 6, 392; M_*sham*_ = 14, 693, SD_*sham*_ = 9, 599), *t*_(33)_ = 1.45, *p* = 0.16, Cohen's *d* = 0.21, while it did not affect response times on believable conclusions (M_*tDCS*_ = 14, 607, SD_*tDCS*_ = 7, 491; M_*sham*_ = 14, 628, SD_*sham*_ = 8, 464), *t*_(33)_ = 0.02, *p* = 0.99, Cohen's *d* = 0.003.

Finally, a three-way validity × believability × stimulation condition was marginally significant, $F_{\left(1,\;33\right)}=3.58,p=0.07,\eta_{p}^{2}=0.10$. Again, because that interaction is only peripheral to our purpose, we refrain from going into details.

## Discussion

The present study explored the possibility that tDCS might decrease the well documented detrimental effect of emotion on analytical reasoning. Results showed that anodal stimulation indeed decreased the detrimental effect of emotion, but only for valid problems. Anodal stimulation had no effect on emotional invalid problems. As it is displayed in Figure 1, anodal stimulation canceled the deleterious effect of emotion on valid problems, while it had no effect for neutral problems. These results are partly consistent with the mechanisms hypothesized to be responsible for the effect of emotion on reasoning: a cognitive load in working memory. However, future studies will need to test this mediation hypothesis directly.

The present results are interesting as they require us to go beyond the initial hypotheses to offer an explanation. If tDCS simply *boosted* working memory, an accuracy boost should have been observed on neutral problems as well as emotional ones. If tDCS *diminished* the emotional reaction to emotional contents, anodal stimulation should have protected participants from the deleterious effects of emotions both for valid and invalid problems.

The observed interaction between stimulation and validity is intriguing, as it suggests that the two mechanisms postulated may interact in interesting ways. Early research on categorical syllogisms, however, has shown that people perform better on valid problems than on invalid problems (see Dickstein, 1975, 1976; Roberge, 1970), as we observed in our results. These differences in accuracy between valid and invalid syllogisms have also been observed with other dependent measures. Participants take longer to inspect the premises and conclusions of invalid problems, compared to valid problems (Stupple and Ball, 2008). Also, people are more confident in their response for valid than for invalid problems (Quayle and Ball, 2000).

Different explanations may account for the observed interaction between stimulation and validity. Some neurostimulation studies have reported interactions between difficulty and stimulation (Schwarzkopf et al., 2011), consistent with the perspective of stochastic resonance (for a detailed account, see Stocks, 2000). According to this hypothesis, information transfer may be enhanced by the addition of mild levels of noise, lowering the response threshold. The level of this threshold differs according to the difficulty of a given task. Studies on sensory signal detection have provided direct evidence for that possibility. It was shown that the effect of transcranial stimulation is determined by an interaction of the stimulation parameters and the strength of the signal: performance related to a weak perceptual signal may be enhanced by adequate stimulation (Abrahamyan et al., 2011). It is clear, however, that this explanation cannot account for our results. This account would have predicted that invalid problems, for which baseline performance is low, would benefit the most from tDCS (possibly because there is more room for the performance to increase). Our results clearly show that it is not the case, as only performance on valid, less difficult problems was improved by tDCS.

Another possible explanation of our results considers the asymmetry between correct responses to valid and invalid problems. In valid problems, the correct response is “Yes” (i.e., the conclusion is logically valid). This is incongruent with the emotionally negative content included in emotional problems. By contrast, the response “No” (i.e., the conclusion is not logically valid) is the correct response to invalid problems. This response is congruent with the negative content displayed in the invalid problems. Considering the important role of the dlPFC in cognitive control (Miller and Cohen, 2001), the asymmetry between yes and not responses might explain why invalid problems may be less *easily* modulated by tDCS.

Our results suggest an effect of anodal stimulation on emotional reasoning. To our knowledge, this is the first demonstration of an effect of tDCS on reasoning. It provides new insights into the debate regarding the generalizability of the effects of tDCS. We showed that tDCS affects a higher-level cognitive task, known to rely on working memory. Our results also show that the effects of tDCS are specific to a condition (emotional contents) that is particularly taxing in terms of working memory resources. Other tDCS studies using offline protocols with montages similar to ours have shown effects on different cognitive tasks. Zaehle et al. (2011) have shown that anodal stimulation of the left dlPFC increased performance on a 2-back task; the only parameter differing with our study being amperage, which was 1 mA in their study (while it was 2 mA in the present study). Hoy et al. (2013) directly examined the effect of dose and duration of tDCS on working memory enhancement. They highlighted that increased doses did not necessarily result in greatest enhancements of working memory and that the greatest effects on working memory were actually observed when stimulation was set at 1 mA (although a 2 mA stimulation still increased performance compared to a sham stimulation). This result is consistent with the observations of Gladwin et al. (2012) and Jeon and Han (2012) who observed effects of 1 mA anodal (offline) stimulations of the dlPFC on working memory performance.

There is no doubt that differences in the parameters used in tDCS research may affect results and need to be considered when assessing comparability between studies. That issue has been documented. Tremblay et al. (2014) reviewed more than 60 tDCS studies, to emphasize both the commonalities and differences (in terms of montage, dose, etc.) between protocols. Of importance is the fact that, among the diversity of studies and the diversity of montages and procedures, all the studies involving *offline* procedures using *1–2 mA* stimulations showed positive effects of anodal stimulation of dlPFC (with the cathode placed on the reference site) on cognitive activities, including activities requiring working memory. We are confident that the growing number of studies using tDCS will contribute to further refining the methods and protocols, increasing the probability of being able to clearly observe when tDCS does and does not have an impact on cognitive activities.

## Ethics statement

This study was carried out in accordance with the recommendations of Comité d'éthique de la recherche avec des êtres humains de l'Université du Québec á Trois-Riviéres with written informed consent from all subjects. All subjects gave written informed consent in accordance with the Declaration of Helsinki. The protocol was approved by the Comité d'éthique de la recherche avec des êtres humains de l'Université du Québec á Trois-Riviéres. (Ethics Certification number: CER-14-09-147-06.04).

## Author contributions

BT designed the experiment, collected the data, analyzed and interpreted the data, and wrote the manuscript. VM-C designed the experiment, collected the data, and proofread the manuscript. J-FL and IB designed the experiment and proofread the manuscript.

### Conflict of interest statement

The authors declare that the research was conducted in the absence of any commercial or financial relationships that could be construed as a potential conflict of interest. The reviewer, LW, and handling Editor declared their shared affiliation.

## Footnotes

^1^That first part of data collection explored how online tDCS may mitigate the detrimental effect of induced stress on a N-back task. Using a 2-back and a 3-back version of the task, we showed that anodal stimulation mitigated the effect of stress (induced with the presentation of loud alarm sounds) on performance. As it is not directly in the scope of the present research, which explores offline tDCS stimulation, we do not detail that first part further.

^2^Additional analyses including stimulation order as an additional independent variable showed that order never impacted any interaction including emotion × stimulation condition (all *F*s < 0.38, all *p*s > 0.54). Our three-way validity × emotion × stimulation condition interaction remained largely significant, *p* = 0.02. As a result, we do not consider order any further.
